# Multi-dimensional constrained energy optimization of a piezoelectric harvester for E-gadgets

**DOI:** 10.1016/j.isci.2021.102749

**Published:** 2021-06-17

**Authors:** Lucas Q. Machado, Danill Yurchenko, Junlei Wang, Giacomo Clementi, Samuel Margueron, Ausrine Bartasyte

**Affiliations:** 1 IMPEE, Heriot-Watt University, Edinburgh, UK; 2 School of Mechanical and Power Engineering, Zhengzhou University, Zhengzhou, China; 3 FEMTO-ST Institute, University of Bourgogne Franche-Comté, Besançon, France

**Keywords:** engineering, mechanical engineering, energy systems, materials science, energy materials

## Abstract

This paper addresses the design optimization process of an energy harvesting device for scavenging energy from an e-gadget, utilizing its ”rocking” motion. The plucking mechanism inspired by the frequency up-conversion technique provides initial displacement exciting piezoelectric beams and increases the total number of excitations multiple times. The harvester is designed in conjunction with the multidimensional surrogate optimization algorithm to maximize the device’s performance considering the geometrical features of the concept and the constrained operating environment. The established numerical model is validated first using a set of experimental data. The obtained numerical results demonstrate that the developed 10.2″ size device produces 55 mJ in half-period when inclined at 45°, which is equivalent to generating 0.3 W. Considering that an iPad of the same size consumes around 3 W, the proposed energy harvester is capable of extending its battery life by 10%.

## Highlights

• The paper provides a novel design of an energy harvesting device for e-gadgets
• Surrogate optimization is carried for global maximization of the device’s performance
• The concept is validated using analytical, numerical, and experimental approaches
• Power generation can reach up to 0.3 W when a 10.2" tablet is considered at 45°

## Graphical abstract

**Figure:**
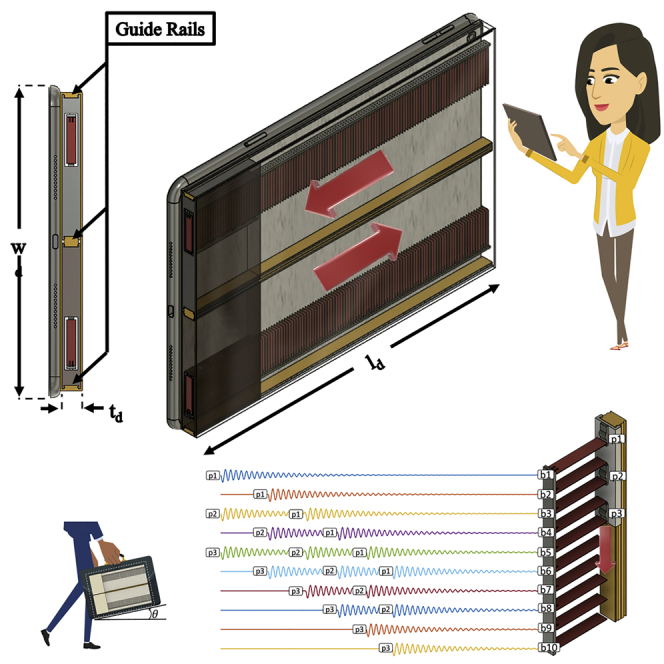

## Introduction

The remarkable progress in miniaturisation of sensors and their low power consumption, achieved at the end of the 20th century, has initiated a wide scientific discussion regarding the new opportunities arising in the area of power generation. The exchange of groundbreaking ideas that started about 20 years ago led to new trends in the development of alternative energy generation technologies, some of which we witness today. The well-established ideas of harvesting solar, wind, water and waves energy from renewable sources, inherited from our ancestors, were complemented by new concepts of energy harvesting from ambient vibrations and vibrations present in man-made machines and structures. Energy harvesting (EH) from mechanical vibrations has become an appealing alternative to recharge batteries or substitute them completely, since sensors’ battery replacement is often inconvenient, expensive and, sometimes, a risky operation, which contributes to the overall high maintenance cost of various structures. Therefore, considerable efforts have been made toward the development of the EH devices capable of harvesting energy from mechanical vibrations in situ, converting it into electrical energy and providing this energy to a solitary sensor or a sensors network. Thus, the development of EH devices has been closely associated with such a paradigm as the Internet of Things, the Industrial Internet of Things, Wearable and Implantable Body Sensor Networks (Khan and Pathan, 2018; Shaikh and Zeadally, 2016).

Unfortunately, the linear oscillatory systems, including beams described by the Euler-Bernoulli theory, have demonstrated relatively high energy conversion efficiency only within a narrow range of their resonant frequency. Thus, such a system under a broadband or random excitation does not reach its optimal performance. Moreover, since vibrations are considered as an adverse effect, they are typically well-mitigated leaving very little energy available for harvesting in many practical applications. This directly leads to a low power output, where it is neither enough to substitute a battery nor sufficient to properly recharge it. On the other hand, operating at its resonance frequency may reduce a harvester’s service life or lead to fatigue failure (Kim et al., 2010; Li et al., 2014). These are the factors that led scientists to focus on new concepts, looking for nonlinear EH for widening the system operating bandwidth, new multi-functional meta-materials for increasing conversion efficiency, and new power management circuits to simplify and enhance the power delivery.

The usage of an array of beams has also been an attractive option not only to widen the operating bandwidth of the system but also to increase the overall power output. Thus, scientists have started investigating the performance of an array of beams and their joint electrical performances. One attempt to handle the narrow frequency band problem was undertaken in the work of Meruane and Pichara (2015) where the authors used an array of piezoelectric beams connected by springs. However, solving the bandwidth problem with beam array leads to the charge cancellation issue. Lien and Shu (2012, 2013) investigated the electrical aspects of connecting an array of piezoelectric harvesters under several interface circuits and tried to address the charge cancellation problem in the following paper (Lien and Shu, 2013). However, connecting each beam to an independent rectifier and all the rectifiers to the same load, as they proposed, is not enough to avoid charge cancellation when phase shift is present. It should be noted that the idea of using beams arrays has mostly been used for forced vibration systems, whereas the free vibration of beams in an array was mainly used for the purpose of EH with a frequency up-conversion technique, which is another interesting solution to increase the overall power output from low non-resonant excitation inputs.

Mechanical frequency up-conversion (MFU) approach takes advantage of a (non) period and (non) resonant primary element, which is sensitive to a low frequency excitation. As the beam and the primary element interact with each other, the beam harvester receives elastic strain energy in the form of initial displacement. This energy is then released as the beam freely vibrates at its natural frequency. This technique was mainly implemented via impact interaction (Abedini and Wang, 2019; Chen et al., 2017; Dauksevicius et al., 2019; Rui et al., 2021), contact plucking (Kathpalia et al., 2017; Kuang and Zhu, 2017) and magnetic contactless plucking (Chen et al., 2019; Kuang et al., 2016; Xue and Roundy, 2017).

In the scope of frequency up techniques, the operating frequency of the cantilever beam is not dependent on the excitation frequency of the primary element which can improve the power output and the bandwidth operation (Asanuma and Komatsuzaki, 2020; Dauksevicius et al., 2019; Fu and Yeatman, 2019; Rui et al., 2021; Zhang and Qin, 2019). In fact, there are a number of ideas that were proposed for utilizing MFU technique in rotating machines (Fuet al., 2021). Basically, the MFU method can be effectively used when the excitation frequency is low, e.g., in applications oriented to harvest energy from ocean waves and human activities.

Most optimization studies in EH deal with power management and geometry issues. Cai and Harne (2019) integrated a genetic algorithm within an analytical model to develop a custom shaped harvester which improves the power output, bandwidth operation, and mechanical robustness. Qin et al. (2019) conducted dimension analysis to optimize the electromechanical coupling coefficient for shear vibrations, and concluded that the ratio of the width to thickness is the leading dimensional factor. Lü et al. (2019) presents a power density scale equation which combines geometric, mechanical, and electrical parameters of the equivalent piezoelectric circuit/standard energy harvester circuit to achieve the maximum output conversion and storage efficiency for piezoelectric nanoribbons. However, here again the optimization is localized and solely reflects a rearrangement of the variables presented in the already consolidated electromechanical formulations. It is a didactic way to understand the relationship between the parameters; however, it is not an optimization with an application oriented procedure. This is clearly noticed as their conclusions have been already well discussed in the literature, i.e., the power output depends on the capacitance of the beam, its frequency, the load resistance to which it is connected, and the input excitation. They also concluded that shorter and thicker harvesters may lead to higher power density, as expected, since the stress will be higher for the same imposed displacement.

Recently, the optimization of different PE beam’s shapes and cross-sections were studied in the work by Hashim et al. (2021) and Peralta et al. (2020). Some other PE EH devices and concepts can be found in the recent review paper by Yang et al. (2018). However, none of them, including those using piezoelectric arrays, address the optimization required in the macro or device level. Optimization approaches in EH have mostly been carried out within the local domain, where material, shape, bandwidth, and circuit configuration were the focus of the analyses. These aspects, although of high importance to understand the electromechanical coupled behavior and improve manufacturing processes, do not necessarily lead to an optimized device as what is optimal for a single beam may not apply for an array of beams. An optimized device is not limited to an optimized beam shape or material, nor to an optimized circuit configuration. It encompasses not only the local electromechanical characteristics but several other interconnected parameters. In the constraint environment of a device, the number of beams, their thickness and tip displacement are all interconnected and, thus, one has to deal with the fine balance of these and other parameters in the available space. To the best of the authors’ knowledge this problem has not yet been addressed in the literature.

Thus, in this paper, the optimization of the entire device within a constrained space is considered for the first time. Besides, the paper proposes a novel plucking concept for engaging beams multiple times based on the predefined weight of the moving mass, the beams’ stiffness and deflection, which allows defining a constant number of excitations within a half-period operation. This concept removes the limitation of exciting each beam only once as the plectrum moves one way forth or back and allows to optimize the weight of the moving mass to which the plectrum is attached. In section 2, the paper presents the novel concept and introduces the main parameters related to the performance of the device. In section 3, the numerical results obtained by the numerical Finite Element (FEM) model, created in Abaqus, and a dynamical model, created in MATLAB, are validated experimentally for a single unimorph beam. Section 4 presents the results of the multidimensional surrogate optimization approach, indicating the power density of the optimized device, its characteristics, and its absolute output power. The Conclusions summarise the findings and propose further developments to expand the device’s application.

### Design concept

The harvester is conceptualized based on the “rocking” motion of an electronic gadget such as a smartphone or a tablet and can be scaled to meet the size variations between gadgets. A moving lumped mass, represented by a moving carriage, is used to convert the potential energy of the device into kinetic energy and then into electrical energy by engaging the PE beams using the MFU concept. Therefore, the harvester takes advantage of free vibrations imposed by the mass on the piezoelectric beams engaging some of them simultaneously.

**Figure 1:**
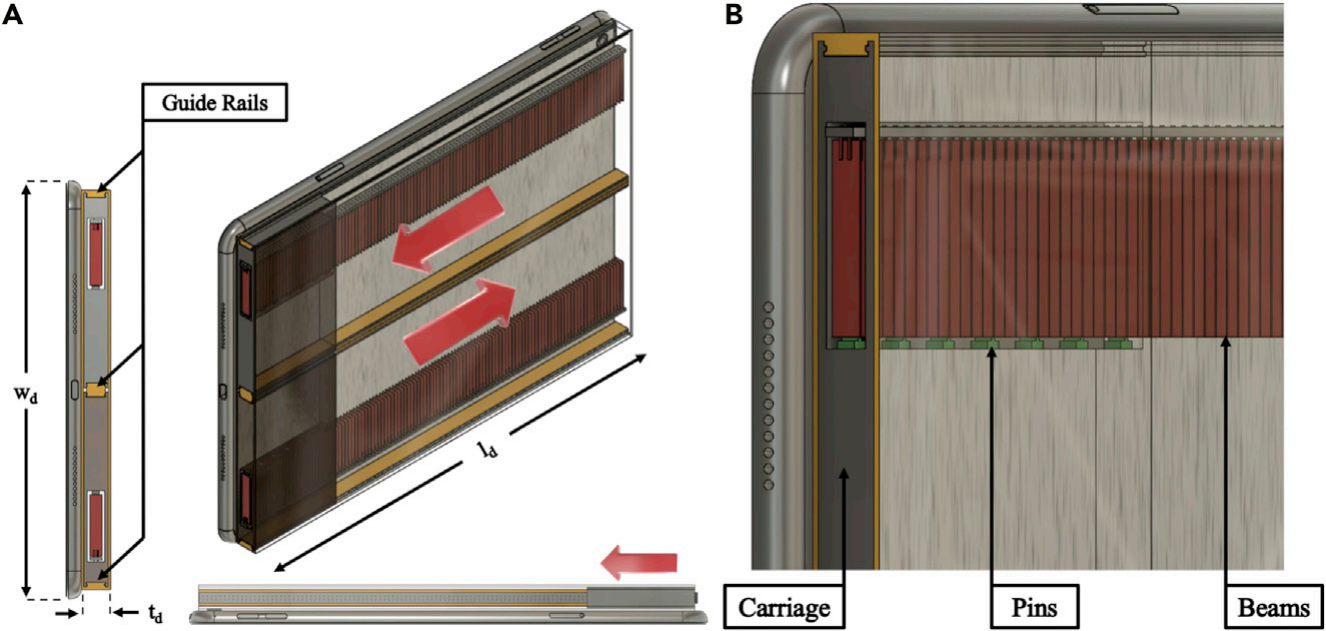
Proposed energy harvester concept: (A) overall view and (B) zoom in view of the beams and pins in the comb-like structure.

Figure 1A illustratesthe design of the harvester, which is cuboid in shape and is divided into two symmetric halves–the top and the bottom–which are identical but operate independently. The length and the width of the harvester match those of the targeted gadget, as demonstrated in the side and top views, whereas the harvester thickness (perpendicular to the plane of the gadget) can differ to accommodate different weights. Each half is comprised of a carriage, which can move along the guide rails, indicated in yellow. The carriage side, facing the free end of the beams, has pins which overlap with the beam, thereby exciting them when passing by, as shown in the zoomed-in image of Figure 1B.

The number of pins directly influences the number of excitations delivered to each beam. The introduction of pins allows the proposed design to move away from from 1-beam 1-excitation option to the 1-beam multiple excitations paradigm, significantly increasing the number of excitations made by the weight. The PE beams of 5 mm-width are placed along the cuboid’s length, facing the direction of the carriage motion so that the beams are plucked by the pins while the carriage moves, as presented in the top and left views of the harvester. The device is designed to work at inclination angles as low as 5°, which significantly reduces the gravity force acting on the carriage and directly affects the minimum required carriage mass.

Thus, having defined the volume of the device, it is important to find the optimal number of pins, the thickness of the beams and their deflection, which, in turn, will require a minimum carriage mass. Knowing the relationship between these parameters one can maximize the amount of the overall output energy. Finding the most efficient parameter combination requires the adaption of an optimization procedure, which is accomplished by a numerical algorithm that simulates the dynamic response of the device under various scenarios. The analytical dynamic model adopted for the algorithm and the FEM element model built to evaluate the response of the beam are validated against experimental data next.

### Models and validation

To estimate the device performance with relatively high number of beams (100–500) it is important to develop a numerical dynamic model of the device. The FEM can accurately predict the single beam behavior, but it is computationally costly to simulate the dynamic response of hundreds of beams using the FEM analysis. Thus, the FEM model is validated first, using the experimental results for the unimorph PE beam. Next, the FEM model is used to simulate the dynamic behavior of a single bimorph beam which is then used to validate the dynamic model of multiple bimorph beams as a device.

The linear constitutive equations that describe the electromechanical model can be derived from the consideration that the variational indicator is zero, according to Hamilton’s principle (Liao and Sodano, 2008; Sodano et al., 2004). It governs the piezoelectric material’s behavior and couples the purely linear-elastic formulation given by Hook’s law to the charge equations of electrostatics, which can be derived from the first law of thermodynamics given the homogeneous quadratic form of the electric enthalpy (Tiersten, 1969).

**Figure 2:**
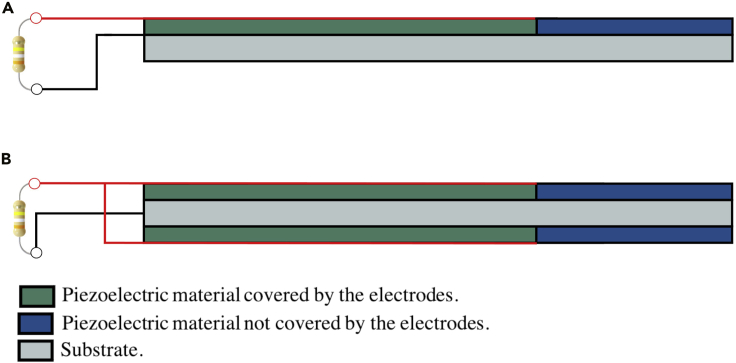
Configuration of the piezoelectric beam (A and B) (A) the unimorph beam and (B) the bimorph beam connected in parallel.

A typical piezoelectric beam structure is composed of one or more piezoelectric layers bonded to a substrate, with or without tip mass, and an electrical interface. Therefore, a piezoelectric energy harvester can be basically described by its natural frequency and damping ratio, which are dependent on the material properties such as the dielectric constant ε, the elastic compliance $s^{E}$, the piezoelectric constant d and density. These are important parameters to determine the electrical potential and power output of the beam harvester. The simplest electric circuit, consisting of a beam harvester connected to a resistive load, is shown in Figure 2.

#### Analytical model

The piezoelectric constitutive behavior is adopted considering a thin beam, based on the Euler-Bernoulli assumptions. The analytical approach, used for the development of the dynamic model in MATLAB, adopts the variational indicator proposed in the works of Liao and Sodano (2008) and Sodano et al. (2004):

(Equation 1) $$\left(VI\right)=\int_{t_{1}}^{t_{2}}\left[\delta K-\delta P+f\delta x\right]dt=0,$$

where *K* is the kinetic energy of the beam, *P* is the potential or elastic energy of the beam, and fδx is the external work applied to the system, having *f* as the external force. Here *P* is defined as the electric enthalpy H(S,E) due to the electromechanical coupling. Next, the definitions of *K*, *P*, and fδx are given by Equations 2, 3, and 4, respectively:

(Equation 2) $$K=\frac{1}{2}\underset{V_{s}}{\int }\rho_{s}{\dot{u}}^{T}\dot{u}dV_{s}+\frac{1}{2}\underset{V_{p}}{\int }\rho_{p}{\dot{u}}^{T}\dot{u}dV_{p},$$

(Equation 3) $$P=\frac{1}{2}\underset{V_{s}}{\int }S^{T}\sigma dV_{s}+\frac{1}{2}\underset{V_{p}}{\int }S^{T}\sigma dV_{p}-\frac{1}{2}\underset{Vp}{\int }E^{T}DdV_{p},$$

(Equation 4) $$f\delta x=\;\overset{n_{f}}{\underset{i=1}{\sum }}\delta u\left(x_{i}\right)\cdot f_{i}\left(x_{i}\right)-\overset{n_{q}}{\underset{j=1}{\sum }}\delta v\cdot q_{j},$$

where *S* and *σ* are the mechanical strain and stress, *E* and *D* are the electrical potential and displacement, *v* and $q_{j}$ are the applied voltage and charge, $u\left(x_{i}\right)$ is the displacement along the position $x_{i}$ of the beam, ρ is the density, and the subscripts *s* and *p* refer to the substrate and piezoelectric materials, respectively. This introduces a set of equations establishing the framework for more specific derivations. Considering a single-degree-of-freedom (SDOF) cantilever equivalent beam model, the constitutive piezoelectric equation is given by (Equation 5) in its most popular strain-charge form:

(Equation 5) $$\begin{array}{l} S_{1}=s_{11}^{E}\sigma_{1}+d_{31}E_{3},\\ D_{3}=d_{31}\sigma_{1}+\varepsilon_{33}^{\sigma }E_{3}, \end{array}$$

where $s_{11}^{E}$ is the compliance under constant electric field, $d_{31}$ is the piezoelectric constants property, and $\varepsilon_{33}^{\sigma }$ is the dielectric constant at constant stress. Therefore, the only non-zero stress is $\sigma_{1}$, implying that bending yields strains in the $1^{st}$-direction only, which polarizes the surface perpendicular to the direction of the applied stress, i.e.,$3^{rd}$-direction. Having defined the constitutive piezoelectric equations, one updates (2), (3) and (4) by incorporating the coupled relationship to them. From these equations, the vibration of the beam is represented in terms of a series of eigenfunctions, presented as:

(Equation 6) $$u\left(x,t\right)=\overset{\infty }{\underset{r=1}{\sum }}\varphi_{r}\left(x\right)\eta_{r}\left(t\right),$$

where $\varphi_{r}\left(x\right)$ is the mass normalized eigenfunction and $\eta_{r}\left(t\right)$ is the modal coordinate of the cantilever beam for $r^{th}$ mode of vibration. For the following steps, it is considered that the beam undergoes strain in x-direction only, represented by $S_{1}$, and polarization in z-direction, represented by $E_{3}$. Therefore, adopting the Euler-Bernoulli beam theory, the strain along the beam is defined as:

(Equation 7) $$S_{1}\left(x,t\right)=-t_{pc}\frac{\partial^{2}u\left(x,t\right)}{\partial x^{2}}=-t_{pc}\frac{d^{2}\varphi_{r}\left(x\right)}{dx^{2}}\eta_{r}\left(t\right),\;$$

where $t_{pc}$ is the distance between the centroid of the beam to the center of the piezoelectric layer. The assumption that the electric potential is uniform across the thickness of the piezoelectric layer ($t_{p}$) allows simplifying the electric field as follows:

(Equation 8) $$E_{3}=-\frac{v\left(t\right)}{t_{p}}.\;$$

Considering the integral of the variational indicator, including mechanical damping through ζ, taking into account the load resistance $R_{l}$ (Sodano et al., 2004), as illustrated in Figure 2, and after some mathematical manipulations, two equations with the electromechanical coupling coefficients are obtained:

(Equation 9) $$\frac{d^{2}\eta_{r}\left(t\right)}{dt^{2}}+2\zeta \omega_{r}\frac{d\eta_{r}\left(t\right)}{dt\;}+\omega_{r}^{2}\eta_{r}\left(t\right)+\alpha_{r}^{eq}\;v\left(t\right)=N\left(t\right),C_{p}^{eq}\frac{dv\left(t\right)}{dt}+\frac{v\left(t\right)}{R_{l}}=\overset{\infty }{\underset{r=1}{\sum }}\alpha_{r}^{eq}\;\frac{d\eta_{r}\left(t\right)}{dt\;},$$

where $\alpha_{r}^{eq}$ and $C_{p}^{eq}$ are the equivalent electromechanical coupling term and capacitance, which depends on the number and configuration of the piezoelectric layers of the beam (see Table 1). N(t) is the forced applied to the beam, which is taken as zero in this paper due to the free vibrations of the beam.

**Table 1:** Equivalent electromechanical coupling term and capacitance for different configurations

Parameter	Unimorph	Bimorph - series	Bimorph – Parallel
$\alpha_{r}^{eq}$	$\alpha_{r}$	$\alpha_{r}$	$2\alpha_{r}$
$C_{p}^{eq}$	$C_{p}$	$C_{p}/2$	$2C_{p}$

Table 1 presents the equivalent capacitance and electromechanical coupling coefficient for unimorph and bimorph configurations assuming that all piezoelectric layers are identical. $C_{p}$ is the capacitance of each piezoelectric layer, which depends on its width ($w_{p}$), length ($l_{p}$), as well as its thickness ($t_{p}$), and $\alpha_{r}$ is the electromechanical coupling coefficient for an unimorph beam. $C_{p}$ and $\alpha_{r}$ are given by the following equations:

(Equation 10) $$C_{p}=\varepsilon_{33}^{S}\frac{w_{p}l_{p}}{t_{p}},$$

(Equation 11) $$\alpha_{r}=-Y_{p}d_{31}w_{p}t_{pc}\frac{d\varphi_{r}\left(x\right)}{dx}{|}_{x_{1}}^{x_{2}},$$

where $Y_{p}$ is the Young’s modulus of the piezoelectric layer at constant electrical field. This is the exact electromechanical equation of the model configuration shown in Figure 2, as presented by Sodano et al. (2004) and Yang et al. (2018), for a piezoelectric beam under transverse vibrations considering the Euler-Bernoulli approach. Some basic steps explaining how to determine this function are given in the works by Sodano et al. (2004) and Yang et al. (2018). Thus, to predict the behavior of a cantilever beam with electromechanical coupling one has to solve Equations 9 with (Equation 10), (Equation 11) and Table 1. The presented analytical dynamic model is developed in MATLAB and validated against experimental and FEM results in the following sections.

#### FEM model and Experimenal study

**Figure 3:**
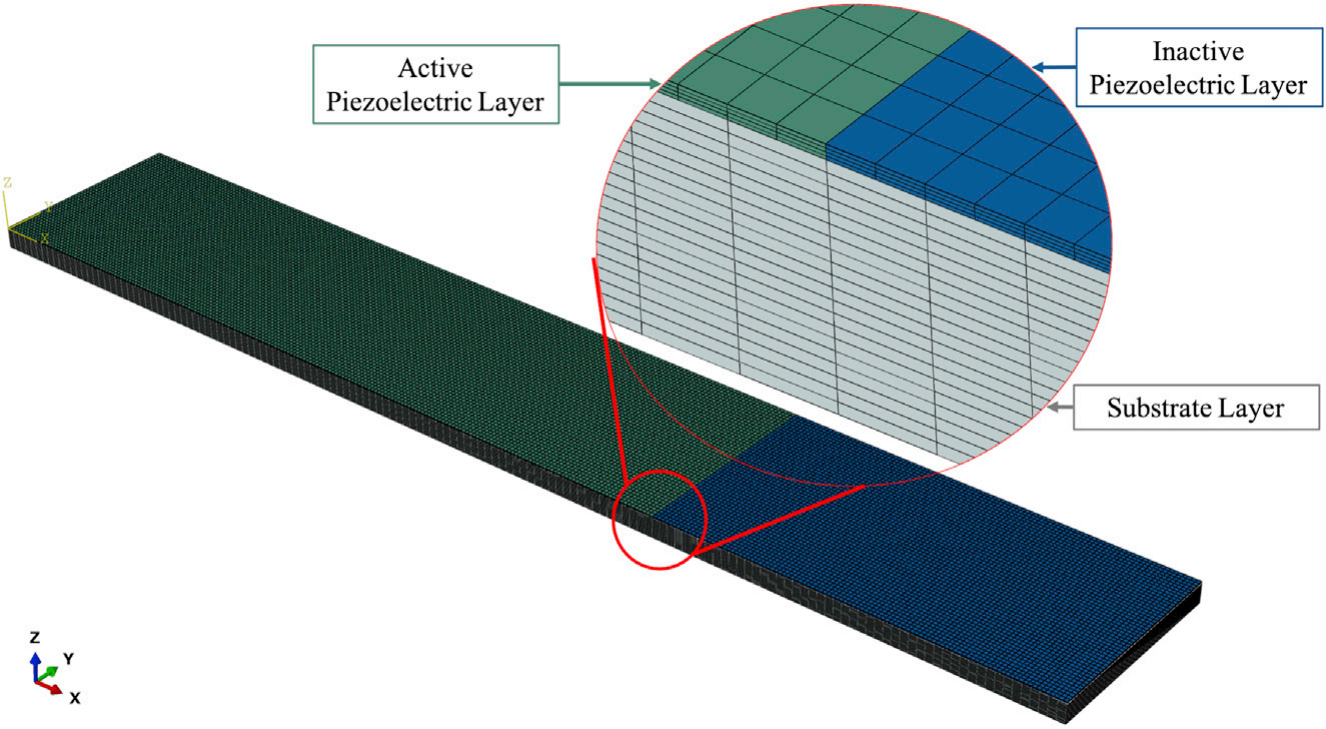
FEM model of the unimorph beam

FEM models of the unimorph and bimorph piezoelectric beams are built in Abaqus. The unimorph FEM model is composed of 40,000 linear piezoelectric hexahedral elements in the active layer and a total of 95,000 linear hexahedral elements in the inactive and substrate layers. For the case of the bimorph model, an identical piezoelectric layer is also attached to the other side of the substrate. Figure 3 shows the mesh utilized in the model of the unimorph piezoelectric beam. The green and blue colors indicate the piezoelectric material, where the green volume represents the part covered by the electrodes while the blue volume is not covered by electrodes and therefore is inactive electrically. The gray volume represents the substrate of the beam harvester. The FEM model of the unimorph beam is validated by comparing the simulation results to those obtained from the experimental tests. When the unimorph model is validated, it is assumed that it can accurately predict the response of the bimorph model, since the only difference is the presence of the same piezoelectric structure on both sides of the substrate. This will allow validating the dynamic model adopted for the bimorph beam, since no experimental data is available for this configuration of the beam.

#### Experimental study

**Figure 4:**
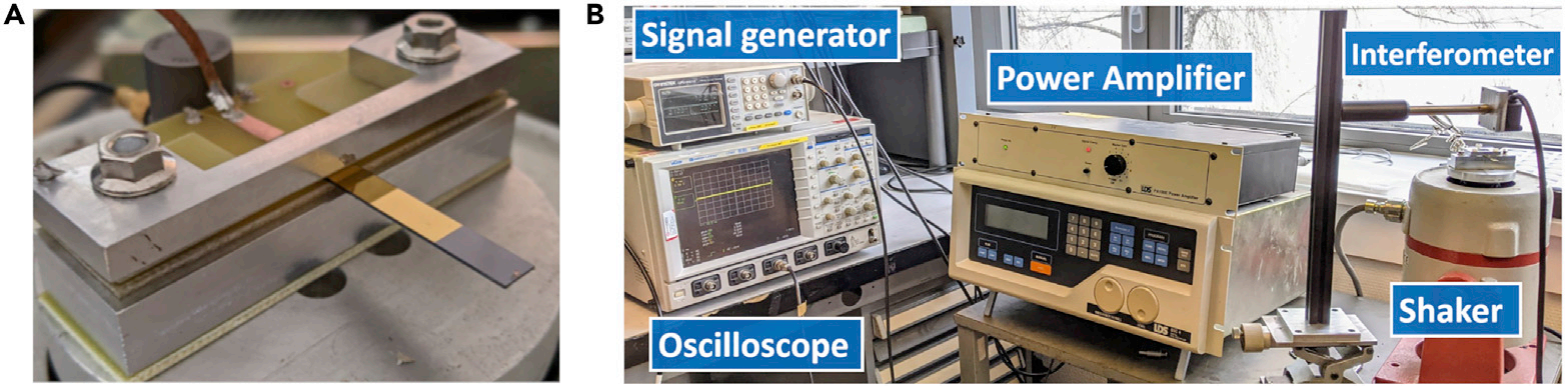
Experimental rig (A and B) (A) LiNbO_3_/Si energy harvester and (B) experimental setup for characterization.

The piezoelectric beams were fabricated exploiting wafer-on-wafer technology, by means of Au-Au bonding between LiNbO_3_ (YXl)/128° (350 μm) and Si (500 μm) 4-inch wafers. This route grants high quality adhesion between the substrate and the active material, minimizing the impact of the adhesion layer to the mechanical properties of the harvester. Both the wafers were sputtered on one surface with Cr/Au adhesion layers, namely 30 nm and 170 nm thick, and then bonded using EVG thermo-compression at room temperature. Afterward, the thickness of the piezoelectric material was reduced to 30 μm in order to tune capacitance and electromechanical coupling of the devices. By means of UV lithography and electron-beam evaporation techniques, Cr/Au top electrodes (30 nm/170 nm) were patterned on the surface of the LiNbO_3_ active layer. In order to optimize the voltage output, just part of the piezoelectric layer was covered with the top electrode, namely 2/3 of the overall length, which follows the same pattern presented by Clementi et al. (2021). Eventually the samples were diced and then cleaned with acetone and de-ionized water. The final device was 30 mm long and 5 mm wide, having 100 mm^2^ of active surface. For this given geometry, we measured the clamped capacitance of the harvester at 1 kHz with a spectrum analyzer (KEYSIGHT E5061B), obtaining C_*p*_ = 1.46 nF. Finally, the samples were wire bonded to a PCB and then clamped on the shaker for testing in harmonic regime (Figure 4A). The nominal dimensions as well as the mechanical and electrical experimental identifications for the energy harvesters used in this study are presented in Table 2.

**Table 2:** Dimensions and properties of the beams

Parameter	Value		Unit
	Substrate Layer	Piezoelectric Layer	
Length, l	30	30	mm
Width, w	5	5	mm
Thickness, t	0.5	0.03	mm
Elastic modulus, Y	169	175	GPa
Density, ρ	2330	4700	$kg/m^{3}$
Piezoelectric constant, $d_{31}$	–	20	pV/m
Relative Permittivity, $\varepsilon_{31}$	–	51	–

**Figure 5:**
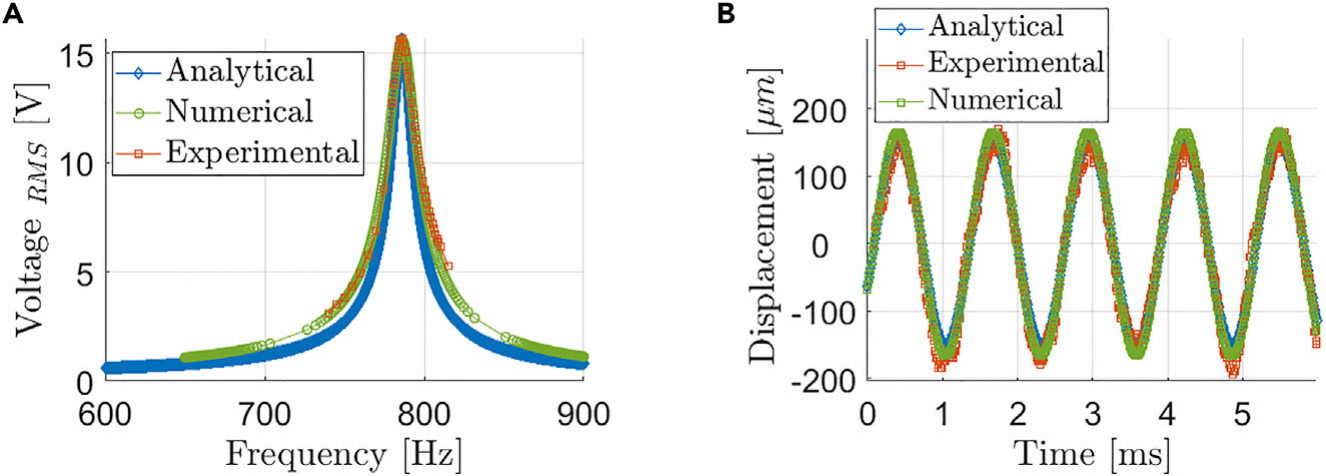
Experimental, analytical and numerical reponses from the unimorph beam (A and B) (A) Frequency and (B) time responses of the unimorph beam: analytical (dynamic model), numerical (FEM) and experimental results at 5.9g excitation level in open circuit.

During the experimental tests, the harmonic sinusoidal input excitation was provided by a signal generator (INSTEK AFG-2012) that was connected to a power amplifier (LDS PA100E) and to an LDS electro-dynamic shaker. An oscilloscope (LECROY LT344) was measuring the output voltage using a 10 MΩ probe, while a laser interferometer (KEYSIGHT E5061B) was measuring the tip displacement (Figure 4B). Several tests were carried out at resonance to evaluate the harvesting potential of the beams, starting with an input acceleration magnitude of 2.5 g up to 5.9 g. For instance, in Figure 5 are presented the experimental results obtained with an acceleration of 5.9 g. With a frequency sweep ranging between 740 Hz and 820 Hz, it was possible to locate the resonance frequency and evaluate the magnitude of the voltage response in the frequency domain. The open circuit resonance frequency of the cantilever was 786 Hz, where we attained V_*RMS*_ = 15.7 V (Figure 5A). Once the resonance peak was identified, we measured the quality factor *Q* of the beam at 3 dB, in order to evaluate the mechanical damping of the structure, obtaining Q=51. Being the base acceleration harmonic, both voltage and displacement showed harmonic response in the time domain as expected. In terms of displacement, we measured 330 μm peak-to-peak in resonant conditions for an acceleration level of 5.9 g (Figure 5B), thus obtaining a force factor of 0.2 mN/V. In particular, the harvester required high acceleration excitation due to the stiffness of the Si substrate and its relatively high resonance frequency. The experimental results concerning frequency and time domain studies were then compared with both analytical and numerical simulations.

#### Models validation

The experiments were conducted in the open circuit configuration, i.e., when there is no current flow, therefore all numerical and analytical results used to validate the model are also presented in the open circuit condition. Figure 5A presents the voltage frequency response for the analytical and numerical models against the experimental data for a forced vibration with input acceleration of 5.9g. The discrepancy in peak value for the voltage output is less than 0.64% when comparing analytical versus experimental data and less than 0.76% when comparing numerical to experimental data. Figure 5B presents the analytical, numerical, and experimental time response for the tip displacement of the beam when a forced vibration with an input acceleration of 5.9g is adopted, indicating the expected electromechanical coupling behavior. The experimental fundamental frequency at open circuit is about 786 Hz and the discrepancy between numerical, analytical, and experimental is less than 1%. Therefore, here it is concluded that both numerical and experimental models are able to represent the dynamic coupled electromechanical behavior of the piezoelectric beam harvester, which will be used to predict the performance of the energy harvester device.

**Figure 6:**
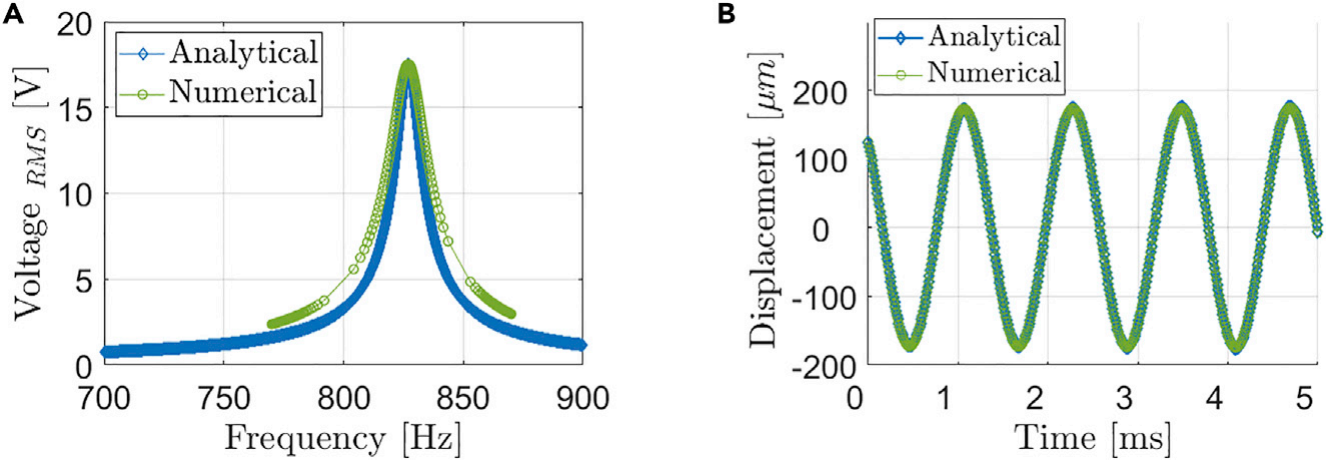
Analytical and numerical reponses from the bimorph beam. (A and B) (A) Frequency and (B) time responses of the bimorph beam: analytical (MATLAB) and numerical forced vibration results at 5.9g excitation level in open circuit.

Now, the bimorph piezoelectric beam is analyzed to allow predicting the performance of the device when it is designed using this beam configuration. In the following analysis, the piezoelectric layers of the bimorph beams are connected in parallel. Since it is an adaptation of the original unimorph beam, there is no experimental data available. Thus, the FEM model for the bimorph beam is used to validate the analytical SDOF model when the second piezoelectric layer is added on the other side of the substrate. Figure 6 shows the frequency response for the analytical and numerical models when an input accelerate of 5.9 g is applied to the system. Both models demonstrate an excellent agreement, having less than 1% discrepancy for the peak values of voltage, displacement, and fundamental frequency at the open circuit configuration. Comparing Figures 5 to Figure 6 it is noted that the bimorph beam has a higher fundamental frequency of 827 Hz, about 5.2% higher than that of the unimorph beam, which was expected due by the addition of a second piezoelectric layer, which makes the beam stiffer.

### System boundaries and excitation mechanism

There are several factors that influence the overall performance of the device, e.g., the mass of the carriage, the displacement applied to the beams, the stiffness of each beam, which is related to its thickness, the number of beams and the distance between them, the number of the pins (to be introduced later) and the distance between them. To address the design of the device, some initial assumptions are made based on the dimensions of a regular tablet available on the market, to which the device is to be attached from the back. Therefore, the length ($l_{d}$) and width ($w_{d}$) of the device are pre-determined based on the size of a gadget $l_{d}\times w_{d}$, where $l_{d}=250.6$ mm, $w_{d}=174.1$ mm, matching the iPad 10.2" dimensions, whereas the device thickness ($t_{d}$), which is equal to the carriage thickness $t_{M}$, is defined a prior and can be changed for a selected device. It is assumed that all beams in the device are identical, undergoing the same tip deflection ($\delta_{b}$). The stiffness of each beam ($k_{b}$) will influence the carriage mass (*M*) as well as the strain $S_{1}\left(x,t\right)$ imposed in each beam, where $x\in \left[0,l_{b}\right]$.

The width of the carriage ($w_{M}$) is defined based on the width of the device and cannot be greater than $w_{d}/2$, since, according to the proposed design, two harvesters can be fitted within the device width. Thus, the mass of the carriage is a function of the carriage length ($l_{M}$), assuming that the carriage is made out of steel with density $\rho =7800\;kg/m^{3}$. The minimum length of the carriage would be that needed to provide enough weight to deflect a single beam when the device is inclined 5^°^ with respect to the horizon. Although this requirement can be changed, influencing the minimum mass of the carriage, the maximum mass of the carriage is limited by the length of the tablet or the amount needed to deflect all the beams at once. The beam’s tip displacement $\delta_{b}$ and the thickness of each piezoelectric beam $t_{b}$ determine the overall space between beams $S_{b}$ in the direction of the deflection, including the free space required for each beam to oscillate. This will determine the total number of beams $n_{b}$ that can be placed along the length of the device, taking into account the limited space available.

Having defined the mass of the carriage one can think about the way the beams are excited by the mass. Typically, when a moving mass is used, it bends a beam once, which limits the total number of excitations to the number of beams $n_{ex}=n_{b}$ as the mass moved from one side of the device to the other. This limitation, however, can be overcome by exciting more beams simultaneously as well as exciting each beam multiple times. This is accomplished by introducing pins/plectrums, which are attached to the carriage and plucks the beams by slightly overlapping them. One way to determine the number of pins $n_{p}$ is by calculating how many beams can be bent simultaneously by the carriage at a given inclination angle and tip deflection. A second way is by deriving the analytical relationship between the number of beams, pins, and excitations and determining the optimal condition. This defines the distance between each pin on the carriage and the number of beams between pins $n_{bbp}$, assuming a symmetrical pins’ distribution along the carriage to account for the carriage motion in opposite directions. It is also assumed that only a single pin can fit between two adjacent beams. In this configuration, the same beam is excited multiple times as the pins move forward with the carriage while the device is being tilted. Thus, the total number of excitations after half a period T/2, defined as the time required for the carriage to go from the elevated end to the other end of the harvester, will be increased.

**Figure 7:**
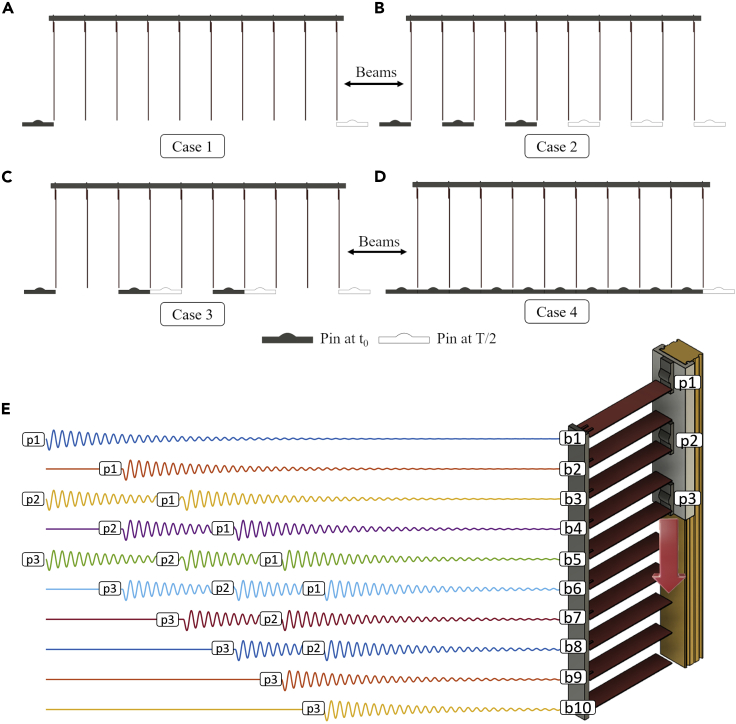
Device’s operation principle (A–D) Cases 1–4 illustrate the concept of pins and beams between pins. (E) engagement of the beams in Case 2 (B).

Figure 7 shows the reasoning behind the pin/plectrum optimal-excitation concept considering an example of 10 beams ($n_{b}=10$) and 3 pins ($n_{p}=3$), with two beams between the pins $n_{bbp}=2$ (Case 2) and three beams between the pins $n_{bbp}=3$ (Case 3). The black and the white pins indicate the initial and final time instances of the pins, moving in the picture from the left to the right. Note that, since the pins are connected rigidly to the same carriage, the distance between them cannot be changed and the mass is allowed to move beyond the first and last beams only by a single pin due to the length restrictions of the device.

Considering these two cases, it is straightforward to count manually the total *calculated* number of excitations, which is $n_{ex}=18$ and $n_{ex}=12$, correspondingly. Indeed, the right pin in Case 2 will excite 6 beams, the middle will excite 6 beams and the left pin will excite 6 beams, which results in 18 excitations in total. Moreover, in the two limiting cases when $n_{p}=1$ (Case 1) or $n_{p}=n_{b}$ (Case 4), the total number of excitation will be equal to the number of beams ($n_{ex}=10$). In Case 1, a single pin will excite all the beams sequentially, whereas in Case 4 each pin will excite each beam simultaneously but only once. Figure 7E demonstrates the beam’s response in Case 3, indicating the motion of each pin plucking the beams. Putting this reasoning into a mathematical expression, the following equation is derived, establishing the relationship between the total number of excitations, beams, pins, and beams between pins:

(Equation 12) $$n_{ex}=n_{p}n_{b}-n_{p}n_{bbp}\left(n_{p}-1\right)\;$$

It should be noted that the number of excitations linearly depends on the number of the beams whereas it has a quadratic dependence on the number of pins. The latter allows finding the optimal number of pins, which is $n_{p}=\left(n_{b}+n_{bbp}\right)/\left(2n_{bbp}\right)$. Thus, for the above case with $n_{bbp}=1$ the optimal number of pins is $n_{p}=5$, which will correspond to $n_{ex}=30$, which is higher then in Case 2. Thus, it can be deduced that with the increase of $n_{bbp}$ the optimal number of pins will go down reducing the total number of excitations. The number of excitations also implicitly depends on all the parameters that influence the number of pins and beams in the device, as previously mentioned. Therefore, the total number of excitations will strongly influence the total energy produced by the device. However, it alone will not determine the best power output scenario as it depends on other variables.

Another important parameter is the thickness of the piezoelectric beams $t_{b}$, which comprises two layers of PE material, which are constant, and the substrate, which is being varied in the analysis, so that $t_{b}=2t_{p}+t_{s}$. Thicker beams are associated with higher natural frequency through the beam’s stiffness $k_{b}$, which, in turn, influences the power output. However, thicker beams require a higher carriage mass to be plucked. Alternatively, it requires a lower number of pins or shorter tip deflection or any equivalent combination of all these parameters. Increasing the thickness also results in increasing the distance between each beam, thereby decreasing the total number of beams in the device. It will impact the total number of excitations within the half-period and will certainly change the total power/energy output. Whether it will be for the better or the worse it is not obvious due to overall nonlinear dependence and inequality constraints. Therefore, applying changes to any parameter requires redesigning the entire device, since all these parameters are interconnected. For this reason, in the designing processes, geometrical and mechanical parameters altogether must be taken into account to determine the best case scenario. Fortunately, all these parameters and their relationship are described by explicit equations, allowing straightforward parametric analysis to be conducted.

In mathematical terms, the above parameters form a set of equations and inequalities. Some inequalities are related to the size of the carriage/mass and the space between pins:

(Equation 13) $$l_{d}\geq l_{M},\;S_{p}\geq S_{b},$$

where $S_{p}$ and $S_{b}$ are the distances between the pins and beams, correspondingly.

The distance between beams and the number of beams are defined as:

(Equation 14) $$S_{b}=2\delta_{t}+t_{b},\;n_{b}=\left\lfloor \frac{l_{d}}{S_{b}}\right\rfloor ,$$

where ⌊⋅⌋ operation indicates the closest small integer, since the number of beams can only be integers.

The number of pins is selected based on the force delivered by a given mass at a given inclination angle and it is assumed to be greater than unity:

(Equation 15) $$\begin{array}{c} n_{p}=\left\lfloor \frac{Mg\sin\theta }{k_{b}\delta_{b}}\right\rfloor >1,\\ k_{b}=3Y_{b}I_{b}/l_{b}^{3},\;I_{b}=\frac{w_{b}t_{b}^{3}}{12}. \end{array}$$

The number of the beams between pins is defined as follows:

(Equation 16) $$S_{p}=\frac{\left(l_{M}-S_{b}\right)}{\left(n_{p}-1\right)};\;n_{bbp}\leq \left\lceil \frac{L_{p}}{S_{b}}\right\rceil .$$

The total number of excitation is given by Equation 12 and the electrical energy generated can be expressed as:

(Equation 17) $$E_{total}=\overset{n_{b}}{\underset{n=1}{\sum }}\left(\int_{t_{1}}^{t_{2}}\frac{V_{n}^{2}\left(t\right)}{R}\;dt\right),$$

where $t_{2}-t_{1}$ is the time required for each beam’s vibrations to decay to 0.85%. Basically, (12) indicates that the contribution of each beam is taken separately and then added together.

**Figure 8:**
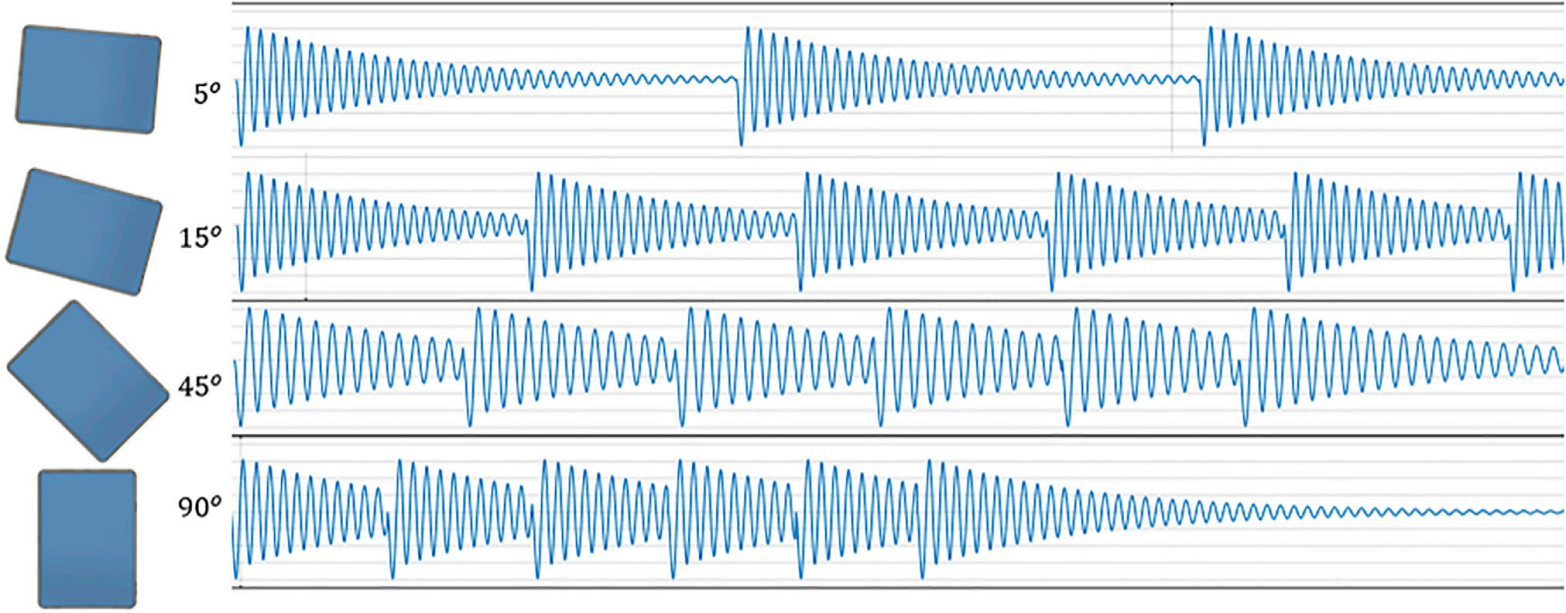
Single beam response at different angles and $n_{p}=6$

It should be noted that (Equation 17) is used because the dynamic behavior of the beams is being changed not only when the inclination angle of the device is varied, but also when the beams at different device’s locations are considered. As can be seen in Figure 8, the higher the inclination angle the greater the effect of the gravity force, moving the mass faster through the beams. Thus, the beams positioned around the central part of the array are excited more times with a shorter interval between the consequent excitations, where the next excitation takes place long before the beam’s oscillations die out.

### Surrogate optimization

Here, the Surrogate optimization is used for global maximization of the objective function defined by the total energy generated by the beam under randomly distributed input parameters. A sweep parametric analysis could be used to investigate the performance of the device under a wide range of case scenarios. The positive aspect of a sweep analysis is that it indicates the optimal values’ space, which helps to minimize the search domain and speed up the computational time when using more robust procedures. However, two limitations pose against this approach. First, a sweep analysis would be a costly process depending on the level of the grid refinement. Second, because the parametric sweep analysis relies on a predefined grid, the optimal set of parameters is likely to lay aside of it, yet within the boundaries defined by it. Due to the boundaries established by the yield stress of the material, the search domain for this analysis has a moderate size, which allows to disregard a pre-optimization sweep analysis. Therefore, the surrogate optimization procedure is adopted (Forrester et al., 2008; Koziel and Leifsson, 2016), as described in Equation 18:

(Equation 18) $$\underset{p^{i}}{\text{max}}E_{T}\left(p^{i}\right)\text{ when }b_{li}\leq p^{i}\leq b_{ui}$$

where $b_{li}$ and $b_{ui}$ are the lower and upper bounds of the $i^{th}$ parameter $p^{i}$.

The surrogate algorithm is used to convert a vector of input parameters p into a scalar output $E_{T}\left(p\right)$. This is accomplished by obtaining a set of output scalars $E_{T}^{i}$ given their respective inputs $p^{i}$ allowing to find the best guess ${\hat{E}}_{T}\left(p\right)$ for the mapping total energy $E_{T}$, generally described as $\left\{p^{i}\to E_{T}^{i}=E_{T}\left(p^{i}\right)|i=1,2,\ldots ,n\right\}$ (Forrester et al., 2008). The surrogate model emulates the original objective function by replacing an expensive optimization by an iterative process that generates a sequence of designs. Thus, the surrogate is a fast and accurate approximation given by this sequence of designs, which is used to emulate the original optimization problem. It is divided in two stages or phases, between which the algorithm alternates. In the first stage, given a predefined boundary, random points are created to assess the objective function and identify the inputs that have notable impact on $E_{T}$. In the second stage, the algorithm searches for a maximum value of $E_{T}$ as it evaluates the objective function. The search is finished when the distance between points are less than the assigned tolerance.

## Results

The analysis is carried out varying the thickness of the device, the thickness of the beams’ substrate (all the beams are assumed of the same structure and size) and its maximum tip displacement. All the other parameters are derived from it, i.e., the number of beams, the number of pins, the distance between beams, the distance between pins, the mass of the carriage, and, more, importantly, the total energy output given by the device. In the 3D space these parameters can be organized to identify how these two parameters influence the overall response of the device. Other parameters influencing the device’s performance, such as the width, the length and the PE layer thickness of the beam, the length and the width of the device, remain constant during the simulation.

**Figure 9:**
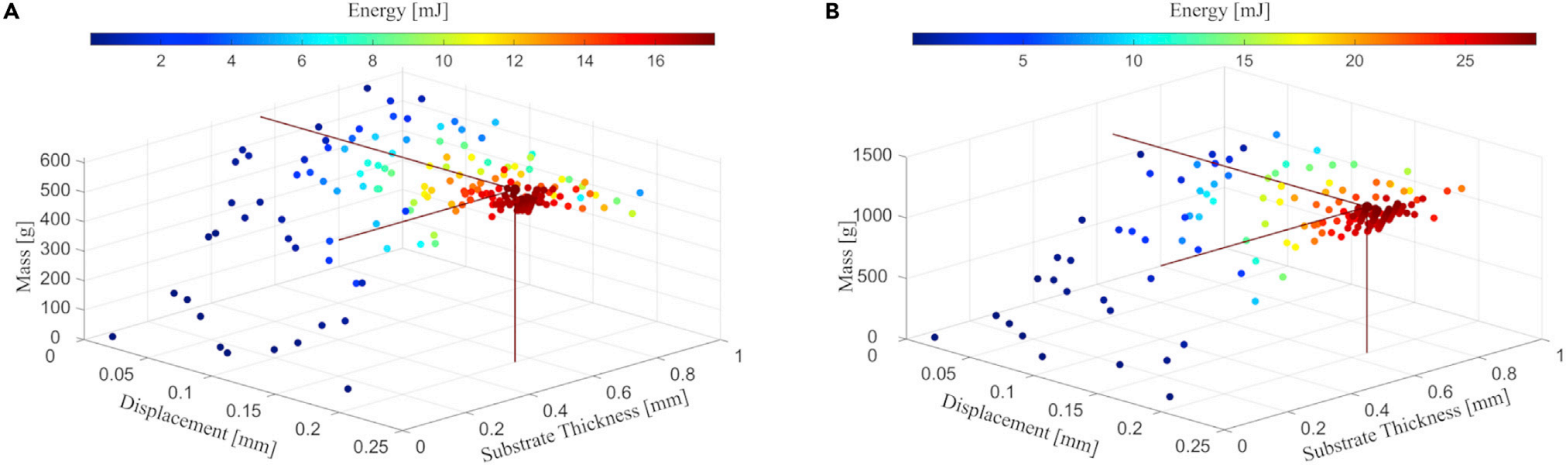
Surrogate optimization analysis for the unimorph beams device at inclination angle of 45° (A) 10 mm and (B) 20 mm thick device.

The first analysis was conducted for the device of $t_{M}=10$ mm thickness consisting of unimorph beams, with the maximum available mass of 1.240 kg and the minimum operating angle of 45°. Figure 9A and Table 3 show the total energy generated from the excitation of all the beams after the carriage has moved across the device in T/2=0.19 sec. In this scenario, the optimal number of beams is $n_{b}=254$, the optimal number of pins is $n_{p}=13$ with the total number of excitations and needed mass equal to $n_{ex}=1742$, $M_{opt}=0.590$ kg, respectively, enabling the device to generate $E_{T}=17.8$ mJ. For the same inclination angle of 45°, Figure 9B and Table 3 show that, by doubling the thickness of the carriage mass, the optimization algorithm suggested $n_{b}=254$, $n_{p}=13$ as the optimal number of beams and pins, respectively. In this scenario the optimal carriage mass of $M_{opt}=1.204$ kg is needed, which will allow generating about $E_{T}=28.2$ mJ.

**Table 3:** Performance of the unimorph beams device using surrogate optimization.

	Device Configuration									Unit
$t_{max}$	10	10	10	20	20	20	30	30	30	*mm*
θ	5°	45°	90°	5°	45°	90°	5°	45°	90°	*o*
$M_{max}$	1.240	1.240	1.240	2.480	2.480	2.480	3.720	3.720	3.720	*Kg*
$M_{opt}$	0.603	0.590	0.590	1.163	1.204	1.179	1.775	1.764	1.724	*Kg*
$l_{M-opt}$	121.8	119.2	119.2	117.6	121.6	119.1	119.6	118.9	116.2	*mm*
$t_{s-opt}$	243	555	645	292	649	798	347	775	847	*μm*
$\delta_{b}$	200	200	200	200	200	200	200	200	200	*μm*
$n_{b}$	372	254	233	347	232	204	322	208	196	–
$n_{p}$	16	13	12	19	17	13	18	15	16	–
$n_{bbp}$	12	10	10	9	7	8	9	7	6	–
$n_{ex}$	3072	1742	1476	3515	2040	1404	3042	1650	1696	–
$E_{T}$	4.9	17.8	21.5	8.3	28.2	33.7	11.4	36.9	43.2	mJ
T/2	0.55	0.19	0.16	0.56	0.19	0.16	0.55	0.19	0.16	S
$P_{d}$	0.015	0.159	0.227	0.013	0.123	0.178	0.017	0.110	0.156	W/kg

Therefore, the optimization algorithm neither simply doubled the number of pins due to the doubled mass allowed nor was the the new proposed configuration able to double the harvested energy. The reason for the first failed expectation is due to (12), which informs that for $n_{b}=254$ and $n_{bbp}=10$, the optimum number of excitation is reached when $n_{p}=13$. If the number of pins were doubled, and the number of beams between beams reduced by half, the total number of excitations would be about 1568, which is less than $n_{ex}=1742$, thus resulting in less energy generated. This is at first counter intuitive, as decreasing the distance between pins and adding more pins can easily mislead someone to think that more excitation will be available. However, due to the quadratic relationship between $n_{p}$ and $n_{b}$, this is not always the case. So, the algorithm indicated an alternative where the thickness of the substrate of the beam can be increased, which pays back by decreasing the number of beams. Ultimately, it is not possible to double the energy output given the doubled mass, but it was possible to increase the energy output by about 69%.

Table 3 summarises the results of different considered scenarios for the device with unimorph piezoelectric beams. In this table, $t_{max}$ is the maximum available thickness of the carriage. It should be stressed that although the devices are called by this maximum thickness, they do not have to be that thick, in fact, the numerical analysis advises how thick the device should be. The device inclinations angle θ, the maximum mass of the carriage available $M_{max}$ and the optimal mass $M_{opt}$ suggested by the numerical algorithm are presented in next two rows. The optimal mass is a function of the optimal carriage length, $l_{M-opt}$, which depends on the number of pins and the distance between them. The beams’ parameters $t_{s-opt}$ and $\delta_{b}$ represent the optimal substrate thickness and displacement achieved by the optimization procedure. The last three rows in the table are the total generated energy $E_{T}$ in a single run, the time T/2 required to complete the single run and the power density $P_{d}=P_{M}/M_{opt}$ ( $P_{M}=E_{T}/\left(T/2\right)$), which gives an indication of the design efficiency. It should be noted T/2 is calculated approximately taking into account the length of the mass and the available distance to move from one side to another.

One can observe from Table 3 that the thickness of the beams’ substrate increases for thicker configurations of the device and for higher minimum inclination angles, which lead to a lower number of beams. Increased thickness for the same applied tip displacement leads to higher stresses in the piezoelectric material as well as higher resonance frequency. Thus, the algorithm shows that the device benefits more from increased stress and higher frequency rather than from adding thinner beams. This is an interesting result since the device could have benefited from having higher number of thinner beams, as the distance between pins could be decreased and the total number of excitations increased for another optimal number of pins. On the other hand, the optimization also shows that this trend has a limit, as it could have selected thicker beams but decided for a thickness below the admissible upper bound, indicating that the present combination of parameters yielded better results.

As expected, higher inclination angles and higher carriage mass due to higher thickness enable the device to generate higher energy output. For the 10 mm thick device, it is possible to generate 4.9 mJ at 5°, 17.8 mJ at 45°, and 21.5 mJ at 90°. The increased energy output for varying angles is related to the effective weight imposed on the beams due to the gravitational force. The same pattern is noticed for the 20 mm and 30 mm thick devices where there is an increased energy output of about 3.23–3.63 times from 5° to 45° and only 1.17–1.20 times from 45° to 90°. However, the mass increase was not reflected in the proportional increase of the energy output, as doubling and tripling the 10 mm thick mass yielded an energy increase of 69% and 133%, respectively, when the device is inclined at 5°. Based on these results, it is more advantageous to simply use two or three 10 mm devices rather than to adopt the 20mm or 30 mm optimized device. However, in those cases the cost will go up since more beam arrays will have to be added.

**Figure 10:**
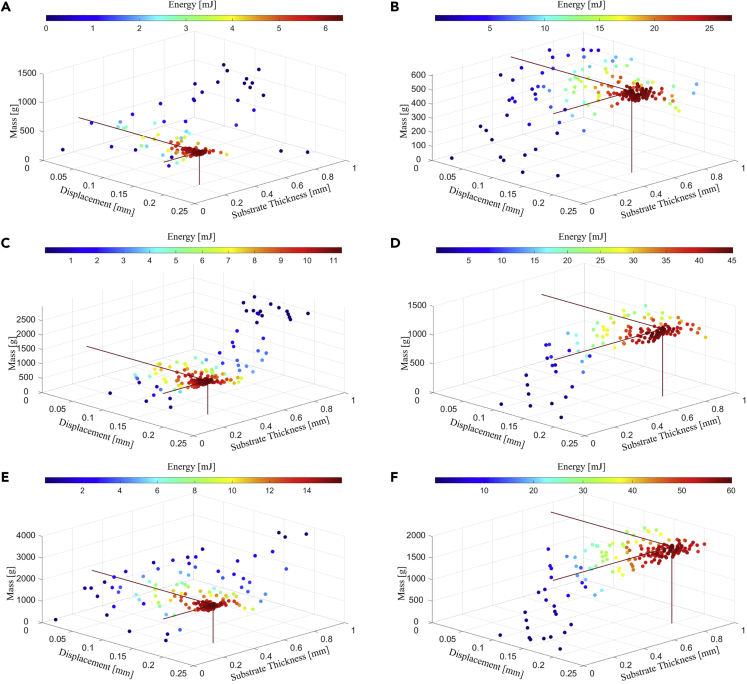
Surrogate optimization analysis for the bimorph beams device with different thickness and inclination angle (A–F) (A) 10 mm at 5°, (B) 10 mm at 45°, (C) 20 mm at 5°, (D) 20 mm at 45°, (E) 30 mm at 5°, and (F) 30 mm at 45°.

Figure 10 presents the results given by the surrogate optimization when the bimorph piezoelectric harvesters are used in the device. This is accomplished by adding another piezoelectric layer of the same dimensions to the other side of the beam. There are six charts where the top, the middle, and the bottom rows correspond to the configurations with the initial thickness of 10 mm, 20 mm, and 30 mm, respectively. The results in the left column of Figure 10 (A,C,E) indicate the device performance at 5° while those in the right column of Figure 10B, 10D, and 10F) demonstrate the device performance at 45°. The results of the surrogate optimization are detailed in Table 4 for the optimal scenario indicated in the Figure 10. It shows that the use of bimorph beams provides significant increase in energy output and this growth is higher for heavier mass and inclination angle. Thus, the minimum gain is for the 10 mm configuration at 5°, which generated 6.4 mJ of energy compared to 4.9 mJ of that for the unimorph, i.e., a growth of 30.6%. On the other extreme, for the 30 mm thick configuration at 90°, there is an increase of 69% when comparing the 43.2 mJ generated by the unimorph beams against the 73 mJ produced by the bimorph beams.

**Table 4:** Performance of the bimorph beams device using surrogate optimization

	Device Configuration									Unit
$t_{max}$	10	10	10	20	20	20	30	30	30	*mm*
θ	5°	45°	90°	5°	45°	90°	5°	45°	90°	*o*
$M_{max}$	1.240	1.240	1.240	2.480	2.480	2.480	3.03	3.03	3.03	*kg*
$M_{opt}$	0.572	0.587	0.587	1.168	1.156	1.176	1.767	1.793	1.784	*kg*
$l_{M-opt}$	115.7	118.8	118.7	118.0	116.9	118.8	119.1	120.8	120.2	*mm*
$t_{s-opt}$	227	524	613	295	653	766	200	787	866	*μm*
$\delta_{b}$	200	199	200	200	200	200	200	200	200	*μm*
$n_{b}$	365	255	233	331	225	204	323	201	189	–
$n_{p}$	13	13	12	14	14	13	18	13	14	–
$n_{bbp}$	14	10	10	12	8	8	9	8	7	–
$n_{ex}$	2561	1755	1476	2450	1694	1404	3060	1365	1372	–
$E_{T}$	6.4	27.4	33.9	11.3	45.4	55.4	15.8	60.5	73.0	mJ
T/2	0.56	0.19	0.16	0.56	0.20	0.16	0.55	0.19	0.16	s
$P_{d}$	0.020	0.246	0.361	0.017	0.196	0.294	0.016	0.177	0.255	W/kg

Within the device with bimorph beams, the same pattern is noticed, i.e., higher inclination angles and higher carriage mass due to its higher thickness enable the device to generate higher energy output. Also, the proportion with which the mass was increased did not reflect the increase in energy output, as doubling and tripling the mass in the case of 5°, yields an increase of 1.76 and 2.47 times, respectively. The mass and the length of the carriage stays within the same range varying about 5% for both devices with bimorph and unimorph beams. However, for most cases, the number of pins is lower and the number of beams between pins is higher for the bimorph, which leads to a lower number of excitations. This takes place mostly because the bimorph beam is stiffer than the unimorph beam for the same substrate thickness. As an example, for the configurations of 10 mm and 5°, the calculated optimal substrate thickness of the unimorph beam (243μm) is 7% higher than that of the bimorph beam (227μm), however the stiffness of the bimorph beam is still 26% higher than that of the unimorph beam due to the doubled piezoelectric layer. Since the optimal mass length of the carriage differs by only about 5% for both cases, the total number of the pins has to be decreased to accommodate the total possible number of simultaneous plucking when it comes to the stiffer beams. The cases where the number of pins and the number of beams between pins are similar, the discrepancy in the stiffness of the bimorph and unimorph beams within the given configuration is not significant.

Coming back to the bimorph device, it can be observed that in all the cases the optimal mass, based on the optimization algorithm, has never achieved its maximum allowed value, but remains around the half of it. One can further compare the influence of the increased mass looking at the devices with 10 mm, 20 mm and 30 mm at 5°. Since there is an optimal number of pins, which is achieved by assuming $n_{bbp}=1$, it directly leads to the optimal length of the carriage mass based on the distance between the beams. In general, this mass can be increased or reduced by changing either its length or its thickness. Increasing the carriage length will reduce the total number of excitations, since it will either allow non-optimal number of pins or reduce the distance traveled by the carriage between the ends of the device, reducing the total number of engaged beams.

Another option to increase the mass is to increase its thickness, which will affect the number of pins, since more beams can be engaged. But this is not necessarily the case, as we can see by comparing 10 mm and 20 mm devices at 5°. In the case of 20 mm device, the beam’s thickness was increased from 227μm to 295μm, as their deflection remained the same. This led to a lower number of beams, one pin was added, and, as the result, the lower number of excitations out of all presented cases. Despite these facts, the 20 mm device was able to generate 76.5% more energy than 10 mm device. Further increase in mass, leading to the 1.505 kg (30 mm, 5^°^) configuration, delivered 2.47 times more energy output than the 10 mm configuration, reaching 73 mJ after T/2. It should be mentioned that in this case the maximum stress was around 60% of the yield stress, according to the conducted FE simulation.

The presented analysis has demonstrated the response of the energy harvester design for three configurations. Note that the results presented so far consider only the single row of beams device due to symmetry, as shown in Figure 1. Thus, the presented results will be doubled for the entire device. Assuming that the inclination angle of 45° is most appropriate while playing, the following results are achieved: 10 mm device with total weight of 1.1 kg will generate 54.8 mJ with an average power of 0.29 W; the 20 mm device with the total weight of 2.31 kg will generate 90.8 mJ with an average power of 0.45 W; the 30 mm device with the total weight of 3.58 kg will generate 121 mJ with an average power of 0.64 W. It should be stressed that lighter device of 1.1 kg will generate 0.423 W at 90^°^ resulting in the highest power density per mass $P_{d}=0.36$ W/kg from all the considered options.

### Additional study: Larger size gadgets and ultimate power

It is of interest to assess how the device’s performance varies when scaled to meet size variations of other e-gadgets. Next, two other e-gadgets, namely iPad 12.9" of 280.6×214.9 mm and Galaxy S7 12.4" of 285×185 mm are considered. As it can be seen, the former is slightly shorter and wider than the latter, however, the overall area of the former is 11% greater. The study is carried out for the three thickness configurations but keeping the 45° inclination angle. The results of the optimization, presented in Table 5, indicate an increase of 29.9%, 34.1%, and 34.6% for the 10 mm, 20 mm, and 30 mm configurations, respectively, when comparing the device scaled for the Galaxy S7 12.4" with that of iPad 10.2". The energy output generated by the device scaled for the iPad 12.9" surpasses the energy generated by the one scaled for the Galaxy S7 12.4" by 16.6%, 11.9%, and 10.6%, respectively for the 10 mm, 20 mm, and 30 mm configurations. However, in terms of power density, the device designed for the Galaxy S7 12.4" outperforms the one designed for the iPad 12.9". When it comes to the thickness configuration, the thinner device proved to be more mass-efficient. For 10 mm thickness configuration, the power density of the entire device for iPad 12.9" is $P_{d}=0.23$ W/kg, which is slightly lower than $P_{d}=0.240$ W/kg for Galaxy S7 12.4". Interestingly, the power density for the Galaxy S7 12.4" device is at the same level as that for iPad 10.2" (see Table 4).

**Table 5:** Harvester performance in larger gadgets environments

	Device Configuration						Unit
	iPad12.9" 280.6 x 214.9 mm			Galaxy S7 12.4"285 x 185 mm			
$t_{max}$	10	20	30	10	20	30	*mm*
θ	45°	45°	45°	45°	45°	45°	*o*
$M_{max}$	1.838	3.676	5.514	1.532	3.064	4.596	*kg*
$M_{opt}$	0.863	1.772	2.675	0.742	1.479	2.238	*kg*
$l_{M-opt}$	131.7	135.2	136.1	138.1	137.6	138.8	*mm*
$t_{s-opt}$	621	744	793	496	681	774	*μm*
$\delta_{b}$	200	200	200	200	200	200	*μm*
$n_{b}$	259	233	224	298	249	231	–
$n_{p}$	12	15	19	19	16	17	–
$n_{bbp}$	11	8	6	8	8	7	–
$n_{ex}$	1656	1815	2204	2926	2064	2023	–
$E_{T}$	41.5	68.3	90.1	35.6	60.9	81.4	mJ
T/2	0.21	0.20	0.20	0.20	0.21	0.20	s
$P_{d}$	0.229	0.193	0.168	0.240	0.196	0.182	W/kg

These results indicate that thicker beams are preferable to having a higher number of beams, to a certain extent. In addition, higher displacement is more advantageous than having thicker beams. The first assertion comes from the fact that although the thinner beams would allow more beams to be used, the energy produced by two thinner beams in a given space is lower than the energy produced by one thicker beam within the same given space. As an example, is better to have one beam with substrate thickness $t_{s}=$ 400 μm with imposed initial displacement of 100 μm than to have two beams with substrate thickness $t_{s}=$ 200 μm with imposed initial displacement of 50 μm each. In the latter case, the stiffness is decreased 5.5 times and the displacement is decreased by half. In this context, the total energy produced can decrease significantly, dropping over 30 times in some cases. At this point it is clear the reason why the optimization chooses thicker beams over a higher number of beams. This trend is noticed in Tables 3, 4, and 5, as the available mass increases, the thickness of the beams also increases. However, again, this is limited as the algorithm in all cases suggested values below the admissible upper thickness limit. In addition to it, evidently, increasing the thickness of the beam is interesting up to a certain point where the displacement can still be imposed to it, whether due to geometric or load restrictions.

**Figure 11:**
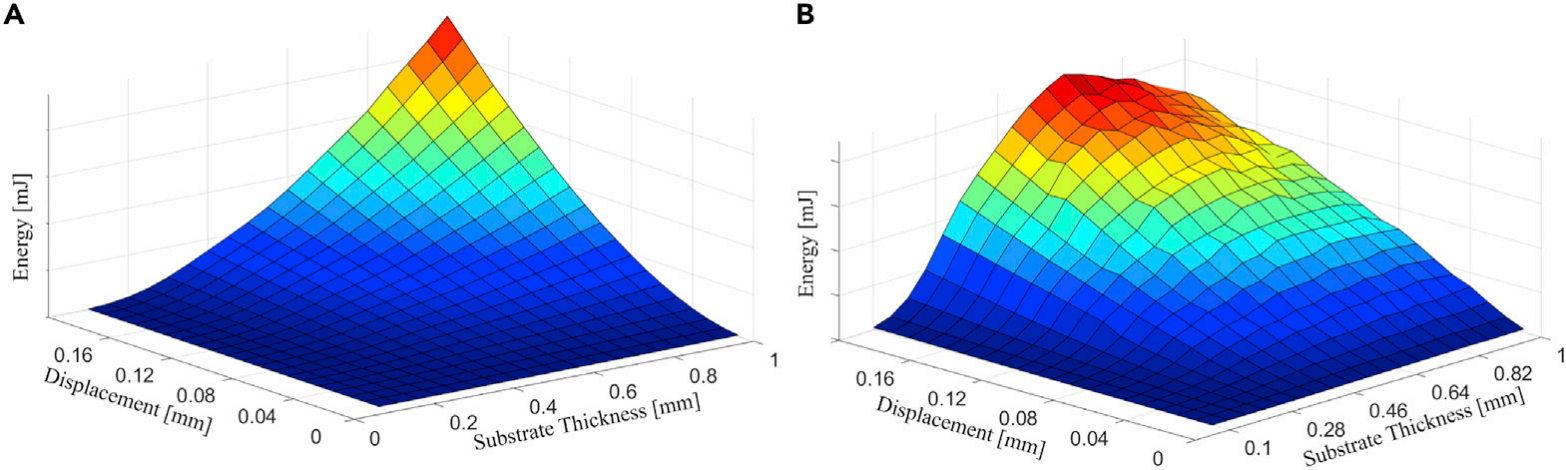
Devices’ performance response change due to the restrictions or constraints imposed on its operation domain Energy output response when (A) no load restriction is applied, and (B) when load restriction is applied.

Within the admissible range of displacements, the algorithm suggested for all the cases that it is always better to adopt its values near the maximum, i.e., 200 μm, rather than to give a space for thicker beams. To analyze how the energy output varies under no load (mass) restrictions, Figures 11A presents the shape of the energy output given admissible values of tip displacement and substrate thickness (energy values were omitted to give emphasis to the shape of the response). It shows that it is better to maintain the driving parameters as close as possible to the upper boundaries. However, when some load restrictions are applied, the response is changed significantly, which is noted in the energy output shape, given the admissible values of the substrate thickness and tip displacement. As can be seen in Figures 11B, the maximum energy output is achieved at the maximum tip displacement but not at the highest admissible substrate thickness. The shape of the chart indicating the devices’ performance changes according to the restrictions or constraints imposed on its operation domain.

## Discussion

This paper proposes a new design for harvesting energy from an e-gadget while playing e-games. Having been attached to the gadget, the harvester utilizes the “rocking” motion of the gadget which drives a mass from an elevated end of the harvester to the lower end. The new concept of multiple pins, introduced and implemented on the mass surfaces, allows plucking the beams multiple times, increasing substantially the overall number of excitations and, therefore, the energy output. The experimental forced vibration tests, conducted with a single unimorph $LiNbO_{3}$ are used to validate both the finite element and dynamic models of the unimorph and bimorph beams.

The paper develops and implements the numerical approach, based on surrogate optimization algorithm, which allows to optimize the harvester based on the gadget size, the thickness and deflection of the beams, and the mass of the gadget. Due to its symmetric design, the harvester, consisting of two identical parts, can generate 0.3 W for a 1 kg harvester, which is around 10% of the power the gadget requires in active operation. The optimization results have shown that the power output does not increase linearly with the increase of the harvester mass, as could be seen by comparing 10 mm, 20 mm, and 30 mm harvesters under the same angle. This fact implies that it is more beneficial to have two half-mass devices than a single device with the equivalent mass.

**Figure 12:**
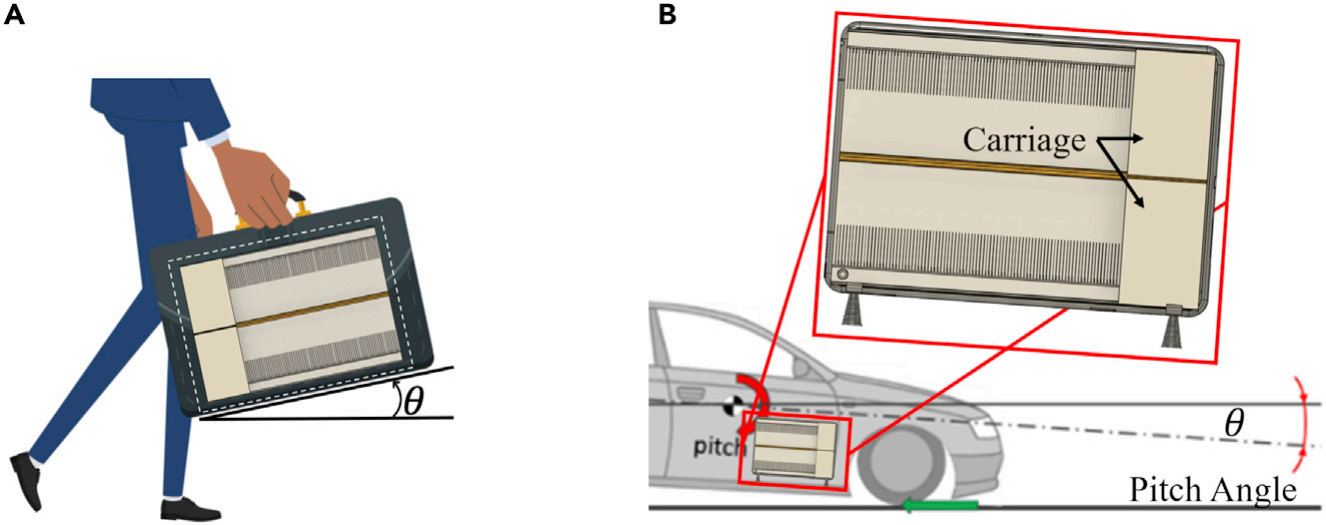
Applications (A and B) (A) Harvester placed in a briefcase (B) Energy harvesting from a pitch motion of vehicle (adapted from Frankenstein, 2017).

The proposed device can also be used in other environments where the changes in the inclination angle of forces are imminent. For instance, the proposed harvester can harness energy when placed in a briefcase held by a walking man. The natural swinging motion of the man’s arm back and forth while walking pitches the briefcase, creating an angle with respect to the horizon, pushing the harvester carriage to move, as demonstrated in Figure 12A.

The device can also take advantage of the pitching moment of a vehicle. Figure 12B illustrates this concept, where the device is placed on the door pocket of the car, although the device can be placed flat on the floor too. Several dynamic phenomena are developed as the car moves and energy can be harvested as the vehicle varies its pitch angle. Basic examples are the changes during acceleration and braking, or while releasing the brake and releasing the accelerator. Thus, there will be a transfer of load from front to rear wheel or vice versa. In either case, the pitch momentum can be used to move the carriage and excite the beams to generate electricity.

### Limitation of study

The authors would like to clarify that the purpose of the paper is to provide a methodology and application for a device-oriented optimization procedure. Thus, the aspects related to the power management circuit for arrays of piezoelectric generators subjected to free vibration is not in the scope of this paper. In this paper, the focus is placed on the role that each parameter plays in the performance of the device, having the total energy generated by all the piezoelectric beams as the leading objective function criterion. The electrical issues encountered at the device level have not been explored in this paper and can be the topic of a separate study.

## STAR★Methods

### Key resources table

**Table:** 

REAGENT or RESOURCE	SOURCE	IDENTIFIER
**Software and algorithms**		

MATLAB	Mathworks	https://UK.mathworks.com/
Abaqus	Dassault Systèmes	https://www.3ds.com/

**Other**		

LiNbO3	https://doi.org/10.1016/j.ymssp.2020.107171	FEMTO-ST Institute

### Resource availability

#### Lead contact

Further information and requests for resources should be directed to and will be fulfilled by the lead contact, Lucas Machado (lq14@hw.ac.uk).

#### Materials availability

This study did not generate new unique reagents.

#### Data and code availability

The data supporting the current study are available from the corresponding author on request.

### Methods

#### E-gadget oriented initial design

First, an initial design is proposed for the target e-gadget. Here, the constraints, the mode of operation, and the conversion mechanism are defined. In the present work, the piezoelectric direct effect is selected as the conversion mechanism in a cantilever beam operating in the bending mode. Given the low frequency nature of the excitation input provided by human activities, the cantilever beams are operated through the frequency up-conversion technique applied through plectrums attached to a moving carriage. The proposed design allows the beams to be excited multiple times by multiple plectrums, governed by a parabolic relationship which offers a optimal correlation between the number of beams within the device and the number of plectrums attached to the carriage.

#### Models and validation

Next, having selected the conversion mechanism, an analytical model is built and validated. To validate the analytical model, an experimental study is conducted for the selected unimorph piezoelectric beam in the cantilever configuration. The two sets of data, analytical and experimental, are compared and the analytical model is validated. To extrapolate the results for the bimorph beam, a numerical model for the unimorph beam is built in Abaqus and validated against the same experimental data provided in the experimental study section. Then, another identical piezoelectric layer is added to the validated numerical model, which now assumes a bimorph configuration. Considering the validation of the unimorph numerical model, the results yielded by the numerical model in the bimorph configuration are used to validate the analytical model built for the bimorph beam.

#### Designing correlations

Having all models validated, a designing algorithm is built establishing the relationship between all constant and variable parameters and constraints. Although the experimental study has been carried out for a specific piezoelectric beam, its geometrical parameters can be turned into variables as long as their combination still allows the beam to behave according to the Euler-Bernoulli theory. For this study, only the substrate thickness is allowed to vary as its influence over the stress is more significant.

#### Optimization procedure

The performance of the harvester device is a function of all the parameters and relationships given in the designing algorithm built for the target e-gadget. Therefore, the MATLAB optimization toolbox is used for global maximization of the objective function defined by the total energy generated by all beams within the harvester. The Surrogate algorithm is selected to perform the task, as it is suitable for expensive objective functions and does not depend on initial estimation. Once the optimization procedure has converged, another analysis is run to verify the solution against other possibilities in the neighborhood of the given parameters.
